# Homologous recombination repair intermediates promote efficient *de novo* telomere addition at DNA double-strand breaks

**DOI:** 10.1093/nar/gkz1109

**Published:** 2019-12-12

**Authors:** Anoushka Davé, Chen-Chun Pai, Samuel C Durley, Lydia Hulme, Sovan Sarkar, Boon-Yu Wee, John Prudden, Helen Tinline-Purvis, Jason K Cullen, Carol Walker, Adam Watson, Antony M Carr, Johanne M Murray, Timothy C Humphrey

**Affiliations:** 1 CRUK/MRC Oxford Institute for Radiation Oncology, Department of Oncology, University of Oxford, Oxford OX3 7DQ, UK; 2 Genome Damage and Stability Centre, School of Life Sciences, University of Sussex, Sussex BN1 9RQ, UK; 3 QIMR Berghofer Medical Research Institute, Brisbane 4006, Australia

## Abstract

The healing of broken chromosomes by *de novo* telomere addition, while a normal developmental process in some organisms, has the potential to cause extensive loss of heterozygosity, genetic disease, or cell death. However, it is unclear how *de novo* telomere addition (*dn*TA) is regulated at DNA double-strand breaks (DSBs). Here, using a non-essential minichromosome in fission yeast, we identify roles for the HR factors Rqh1 helicase, in concert with Rad55, in suppressing *dn*TA at or near a DSB. We find the frequency of *dn*TA in *rqh1Δ rad55Δ* cells is reduced following loss of Exo1, Swi5 or Rad51. Strikingly, in the absence of the distal homologous chromosome arm *dn*TA is further increased, with nearly half of the breaks being healed in *rqh1Δ rad55Δ* or *rqh1Δ exo1Δ* cells. These findings provide new insights into the genetic context of highly efficient *dn*TA within HR intermediates, and how such events are normally suppressed to maintain genome stability.

## INTRODUCTION

DNA double-strand breaks (DSBs) are potentially lethal lesions if unrepaired, and their misrepair can give rise to chromosomal rearrangements, a hallmark of cancer cells (1,2). To maintain both viability and genome stability in response to such lesions cells have evolved two types of DSB repair pathways: non-homologous end joining (NHEJ) and homologous recombination (HR). During classic non-homologous end joining (C-NHEJ), the broken ends are bound by the Ku70/Ku80 heterodimer, and following the removal of damaged bases, are ligated together through the activity of Ligase 4 (Lig4) (reviewed in (3)). During HR repair, homologous sequences within a chromatid or chromosome are used as a template for accurate repair. HR repair is initiated by nucleolytic resection of the 5′ end to generate a 3′ single-stranded DNA (ssDNA) overhang. Resection is a two-step process, which is initiated by the MRN complex (comprised of Mre11–Rad50–Nbs1 in *Schizosaccharomyces pombe* (*Sp*) and in *Homo sapiens* (*Hs*)), and CtIP resulting in partly resected intermediates. During the second step, Exo1 together with Rqh1*^Sp^* (BLM*^Hs^*) facilitates extensive resection (4–6) (reviewed in (7)). The 3′ ssDNA overhang is bound by Replication Protein A (RPA), which facilitates binding of the mediator Rad52*^,Sp^* and removal of secondary structures (8,9). Rad52*^Sp^*, together with the auxiliary heterodimers Rad55*^Sp^*–Rad57^*S*^*^p^* or Swi5^Sp^–Sfr1*^Sp^* mediate the loading of the RecA homologue, Rad51*^Sp^* onto the ssDNA overhang to create a Rad51 nucleoprotein filament. This structure facilitates a homology search and strand exchange between the broken end and the homologous sequence to form a displacement-loop (D-loop) structure (10–13). Following DNA synthesis the invading strand can be expelled by BLM and RecQL5 in mammalian cells, thus facilitating synthesis-dependent strand annealing (SDSA). Alternatively, second-end capture and ligation can result in a double-Holliday junction structure, which can be resolved with or without crossovers, a process involving Yen1, Mus81*^Sp^*–Eme1*^Sp^*, or dissolved through the activities of BLM-Top3 (reviewed in (14)).

Consistent with multiple roles in HR-dependent DSB repair, the RecQ family of DNA helicases plays a key role in maintaining genome stability in all organisms (15). A hallmark of BLM mutations in human cells is increased levels of sister chromatid and inter-homolog exchanges (16). In fission yeast, loss of the BLM orthologue, Rqh1, results in increased genome instability and sensitivity to DNA damaging agents (17,18). Rqh1 is an ATP-dependent 3′ to 5′ helicase, in which the N-terminus interacts with Top3 (19,20). Rqh1 has been implicated in a variety of processes including HR, both before and after Rad51 filament formation (19,21,22); suppressing mitotic crossovers and promoting meiotic crossovers (23–25); suppressing inappropriate recombination following S phase arrest (17,18); facilitating the repair of collapsed replication forks (26–28); intra-S checkpoint function (29); efficient chromosome segregation (30) and telomere maintenance (31,32). A role for Rqh1 has also been identified in regulating HR-dependent Alternative Lengthening of Telomeres (ALT) pathway in the absence of Taz1 in fission yeast (33).

While normally repaired by the NHEJ or HR pathways, broken chromosome ends can sometimes be ‘healed’ as a result of telomeric capture or *de novo* telomere addition (*dn*TA). While *dn*TA is part of the normal developmental process in unicellular ciliates, chromosome healing in mammalian cells is associated with terminal deletions and genetic disease (34,35). Indeed, chromosome healing of a break within the body of a chromosome would be expected to result in extensive loss of heterozygosity (LOH) or potentially cell death through loss of genetic material centromere-distal to the break site. Accordingly, *dn*TA is not normally observed in response to ionizing radiation (IR) or enzyme-induced DSBs in yeasts or mammals (36–38), and may reflect the absence of telomeric seed sequences close to the break site, low levels or inhibition of telomerase, or competition with DSB repair pathways (39–41).

Here, we have investigated the relationship between DSB repair and loss of heterozygosity arising through *dn*TA. By introducing a site-specific DSB into a non-essential minichromosome, Ch^16^, we have uncovered a critical role for Rqh1 helicase, together with Rad55 in suppressing chromosome healing through *dn*TA at break sites. Further analysis suggests that stabilized HR intermediates are efficient substrates for *dn*TA.

## MATERIALS AND METHODS

### Yeast strains, media and genetic methods

All *S. pombe* strains were cultured, manipulated, and stored as previously described (42). A list of strain genotypes can be found in Supplementary Table S1.

### DSB assay

The DSB assay using Ch^16^–MGH was performed as previously described (41,43). The minichromosome (Ch^16^) is a mitotically and meiotically stable 530 kb chromosomal element derived from ChIII (44). The DSB assay was performed at 25°C for strains containing the cold-sensitive mutant *pfh1-R20_cs_* (45) and appropriate comparison strains as indicated in the tables. The colony percentage undergoing NHEJ/SCR (ade^+^ G418^R^ his^+^), GC (ade^+^ G418^S^ his^+^), Ch^16^-MGH loss (ade^−^ G418^S^ his^−^), or LOH (ade^+^ G418^S^ his^−^) were calculated. LOH in this context refers to events which retain the ade^+^ marker but have lost the G418^S^ marker. It is not possible to distinguish genetically between minichromosome loss and other rearrangements resulting in ade^−^ G418^S^ his^−^ colonies, such as isochromomosome formation, using Ch^16^-MGH so this population is collectively termed here ‘Ch^16^ loss’. Each experiment was performed three times using independently derived strains for all mutants tested. More than 1000 colonies were scored for each time point. Mean ± SEM values were obtained from triplicate strains. Differences were deemed significant if *P*-values obtained using Student's *t* test were ≤0.05.

### Pulsed field gel electrophoresis (PFGE)

The PFGE protocols used in this study have been previously described (42). For higher resolution separation of Ch^16^-MGH, a 1.2% chromosomal grade agarose gel was used under the following conditions: 4 V/cm 112° angle with a switch time of 1 min. Samples were separated for 48 h in 1× Tris–acetate–EDTA at 14°C.

### PCR assay for *de novo* telomere addition

Up to 20 randomly chosen ade^+^ G418^s^ his^−^ (LOH) colonies from each genetic background indicated were screened for telomeric sequence distal to the *MAT*a break site as described. PCR amplification with primers targeted to the *rad21* gene (5′-GATTTAAACCTGGATTTGGGC-3′) and telomeric repeats (5′-CTGTAACCGTGTAACCGTAAC-3′) was performed, followed by digestion with *Mfe*I, yielding a distinct 300 bp band in telomere-positive strains.

### Rapid DSB-induction

Strains encoding *urg1::hph* were generated and *urg1::HO* containing strains subsequently generated by cassette exchange as previously described (46). Strains were grown at 32°C in 500 ml of pombe minimal glutamate media (PMG) containing G418 (200 μg/ml), leucine and arginine (100 μg/ml) but lacking adenine, uracil and histidine (47). To induce *urg1::HO* expression, cultures were grown to an OD_595 nm_ of 0.3–0.5. Cells were harvested, washed with water and suspended in PMG containing leucine, adenine, histidine, arginine (100 μg/ml) and uracil (250 μg/ml). 50 ml samples were harvested, washed in water with 0.5% sodium azide then stored at −80°C.

### Gene targeting

Plasmid pJK148 (48) was linearized with NdeI restriction, and transformed into the strains indicated the using Lithium Acetate protocol (47), and the number of Leu^+^ transformants determined for each strain. The gene targeting efficiency was adjusted according to the relative transformation efficiencies of each strain, as determined using a circular pREP81X (49) as a control.

## RESULTS

### Rqh1 suppresses loss of heterozygosity in *rad55Δ*

**Figure 1.**
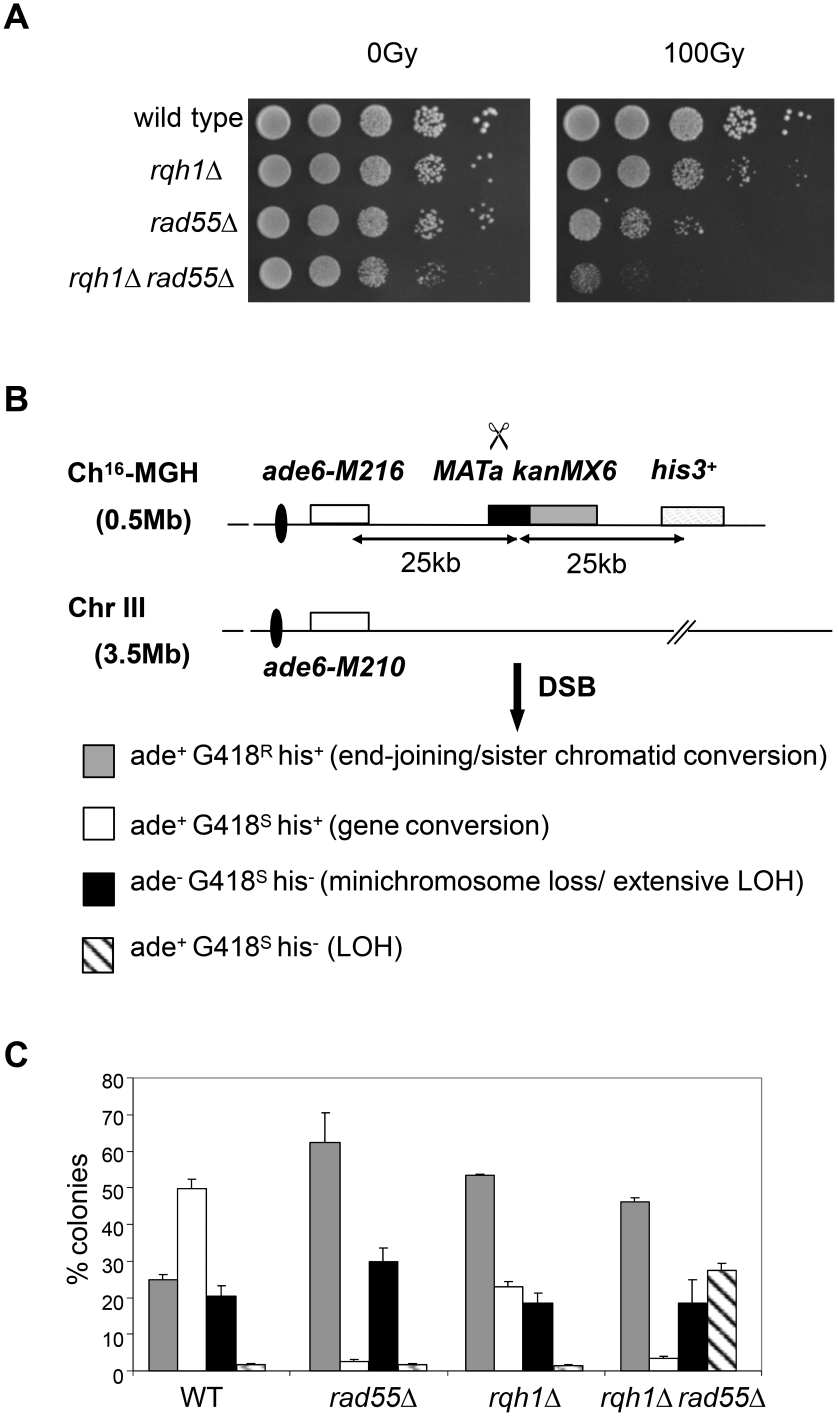
Rqh1 suppresses break-induced LOH in a *rad55Δ* background. (**A**) Spot dilutions of wild-type (TH1900) *rad55Δ* (TH1760) *rqh1Δ* (TH1807) and *rqh1Δ rad55Δ*strains (TH2136) strains grown on YES plates following exposure to 0 Gy, or 100 Gy IR, as indicated. (**B**) Schematic of the Ch^16^-MGH strain and ChIII as previously described. The loci of the centromeres (black oval), *ade6-M216* and *ade6-M210* alleles (white boxes), *MAT*a target site (black box), *kanMX6* gene (gray) and *his3^+^* gene (striped) are as indicated. pREP81X-HO generates a DSB at the *MAT*a target site (scissors). The expected marker loss profiles associated with different repair outcomes are indicated. (**C**) Site-specific DSB repair profile of wild-type (TH1900), *rqh1Δ*, *rad55Δ* and *rad55Δrqh1Δ* strains following HO-endonuclease induction for 48 h. Data are derived from Table 1.

To investigate the role of Rqh1 in genome stability, we examined the relationship between Rqh1 and other DNA recombination genes in the cellular response to DSBs. We found that deletion of *rqh1*^+^ in a *rad55Δ* background resulted in a significant increase in the IR-sensitivity of *rad55Δ* (Figure 1A). To investigate this further, we examined the relationship between *rad55*Δ and *rqh1Δ* deletion mutants using a site-specific DSB assay. Using this assay, different repair and misrepair events can be quantitated by determining genetic marker loss following HO endonuclease induction of a site-specific DSB at the *MATa* site inserted within a non-essential minichromosome, Ch^16^-MGH, derived from chromosome III (Figure 1B). Ch^16^-MGH carries an *ade6-M216* heteroallele which complements the *ade6-M210* heteroallele on ChIII to confer an ade^+^ phenotype through intragenic complementation (50). Following HO endonuclease expression from a thiamine-repressible *nmt* promoter (rep81X-HO) DSB induction can result in a variety of outcomes: DSB repair through NHEJ or sister chromatid recombination (SCR) in which a broken chromatid uses its unbroken sister chromatid as a repair template; failed DSB repair with loss of the minichromosome; gene conversion using the homology of chromosome III; extensive loss of heterozygosity, resulting through break-induced non-reciprocal translocations or partial loss of heterozygosity (Figure 1B) (41).

Surprisingly, HO endonuclease-induced cleavage at the *MAT*a site in an *rqh1Δ rad55Δ* double mutant resulted in a striking increase in LOH (27.3%, *P* < 0.001) compared to wild type (1.7%). This increase in LOH was associated with significantly increased NHEJ/SCR (46.1%, *P* < 0.001) and significantly reduced GC (3.3%, *P* < 0.001) compared to wild type, while Ch^16^ loss (18.6%) was similar to both single mutant and wild-type backgrounds (Figure 1C: Table 1). These findings indicate that Rqh1 suppresses LOH in a *rad55Δ* background. No loss of viability was observed in these strains following DSB induction (Supplementary Figure S1).

**Table 1.** Suppression of LOH by chromosome healing in HR mutant backgrounds

Ch^16^-MGH in genetic background (strain No.)	% ade^+^ G418^S^/ Hyg^S^ his^+^ (GC)	% ade^+^G418^R^/ Hyg^R^ his^+^ (NHEJ/SCR)	% ade^−^ G418^S^/Hyg^S^ his^−^ (Ch^16^ loss)	% ade^+^ G418^S^/ Hyg^S^ his^−^ (LOH)	% ade^+^ G418^S^/ Hyg^S^ his^−^ (*dn*TA)	*P-value* (LOH relative to wild type)	*P-value* (LOH relative to *rad55Δ*)
Wild type*	49.7 ± 2.6	25.0 ± 1.4	20.5 ± 2.6	1.7 ± 0.3	0.0% (0/22)	1.000	
rad55Δ*	2.9 ± 0.7	62.8 ± 9.8	30.5 ± 10.9	1.8 ± 1.1	1.4%(16/20)	0.936	
*rqh1Δ*	22.8 ± 1.4	53.3 ± 0.4	18.6 ± 2.7	1.5 ± 0.3	0.3% (4/20)	0.615	
*rad55Δ rqh1Δ*	3.3 ± 0.7	46.1 ± 1.3	18.6 ± 1.6	27.3 ± 2.1	24.7% (19/21)	3.3 E–06	4.8 E–05
*rad57Δ*	3.8 ± 0.9	73.1 ± 6.0	21.4 ± 5.7	1.8 ± 0.7	0.9% (10/20)	0.955	
*rad57Δ rqh1Δ*	2.0 ± 0.7	76.8 ± 2.1	13.5 ± 3.3	7.7 ± 0.17	3.9% (10/20)	9.75E–06	
*rad55Δ rqh1-K547A*	3.2 ± 0.7	46.7 ± 7.0	22.9 ± 7.6	22.5 ± 5.0	20.3% (18/20)	0.001	0.002
*rad55Δ rqh1*(ΔN1–322)	2.1 ± 0.4	68.6 ± 3.3	17.4 ± 1.8	11.9 ± 1.1	6.0% (10/20)	0.008	3.6 E–04
*Wild type* (25°C)	28.5 ± 1.3	50.9 ± 1.6	10.2 ± 0.8	0.8 ± 0.2	0.0% (0/6)	0.567	
*rad55Δ* (25°C)	6.6 ± 3.7	47.4 ± 11.2	43.9 ± 11.3	0.6 ± 1.1	0.5% (15/20)	0.867 (WT@25°C)	
*pfh1_cs_*(25°C)	16.5 ± 1.7	63 ± 10.5	6.5 ± 2.0	0.6 ± 0.3	0.1% (1/9)	0.609 (WT@25°C)	
*rad55Δ pfh1_cs_*(25°C)	0.9 ± 0.7	72.7 ± 4.2	20.0 ± 2.6	6.4 ± 1.3	4.2% (13/20)	0.013 (WT@25°C)	0.027 (@25°C)
*srs2Δ*	26.1 ± 2.7	49.9 ± 3.6	12.2 ± 2.5	0.2 ± 0.1	0.0% (0/18)	0.006	
*rad55Δ srs2Δ*	7.2 ± 1.3	43.0 ± 9.8	36.4 ± 12.1	2.1 ± 0.4	1.7% (16/20)	0.402	0.654
exo1Δ*	52.7 ± 1.0	33.1 ± 0.7	13.0 ± 0.8	1.1 ± 0.5	0.5% (10/22)	0.233	
*rqh1Δ exo1Δ*	3.7 ± 1.7	54.3 ± 5.8	14.4 ± 6.6	20.7 ± 5.1	12.4% (12/20)	0.004	0.002
*rqh1Δ rad55Δ exo1Δ*	7.5 ± 5.5	58.7 ± 13.5	28.1 ± 18.3	2.7 ± 0.8	0.0% (0/20)	0.209	0.317
rad51Δ*	1.0 ± 0.5	35.9 ± 2.9	57.0 ± 2.9	0.8 ± 0.3	0.6% (20/25)	0.058	
*rqh1Δ rad51Δ*	3.1 ± 0.2	76.8 ± 3.4	19.5 ± 3.3	0.39 ± 0.07	0.3% (15/20)	0.007	0.09

The mean ± SE from at least three independent experiments with three individual strains are shown. % *dn*TA was calculated by multiplying the fraction of *dn*TA positive colonies identified from the 20 ade+ G418S/HygS colonies examined (indicated in brackets) by the % LOH. * denotes values as previously described (Cullen *et al.*, 2007), shown here for comparison.

### Rqh1 suppresses *de novo* telomere addition in a *rad55Δ* background

**Figure 2.**
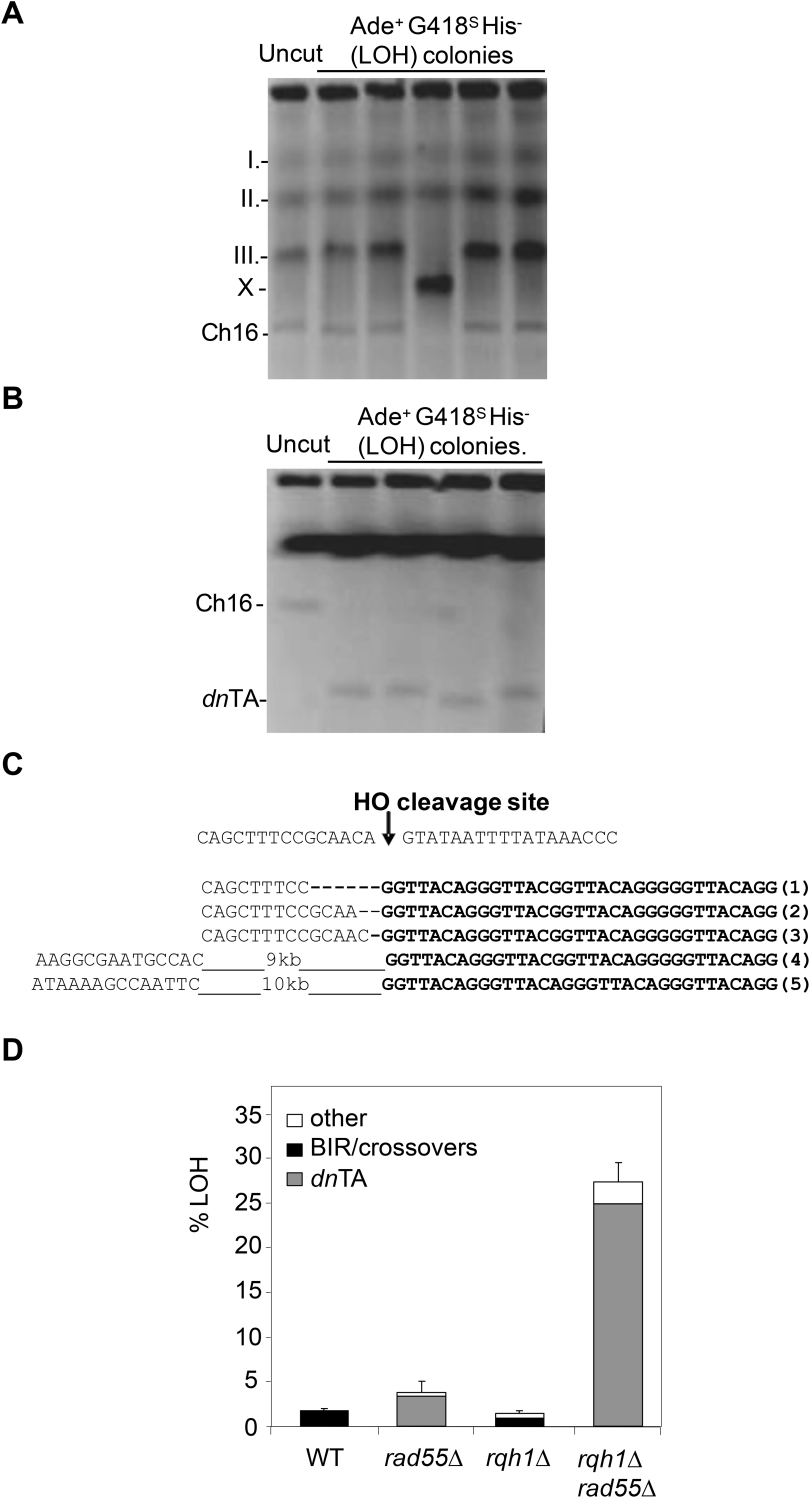
*De novo* telomere addition causes LOH in an *rqh1Δ rad55Δ* background. (**A**) Pulsed Field Gel Electrophoresis (PFGE) of LOH colonies obtained after HO induction in the *rqh1Δ rad55Δ* background. (**B**) High resolution PFGE of LOH colonies. (**C**) The sequence of the HO endonuclease cleavage site within *MAT*a is shown, together with representative genomic DNA sequence data of the region surrounding the *MAT*a site from five individually isolated ade^+^ G418^S^ LOH colonies with truncated minichromosomes, indicating the presence of *de novo* telomeres. (**D**) Graph depicting mechanisms of LOH in wild type (WT, TH1900), *rqh1Δ*, *rad55Δ* and *rqh1Δ rad55Δ* backgrounds following DSB induction in Ch^16^-MGH. Data are derived from Table 1.

To identify the mechanism of break-induced LOH, the chromosomes of 21 LOH colonies from an *rqh1Δ rad55Δ* background were examined by pulsed-field gel electrophoresis (PFGE). While endogenous chromosomes I and II derived from these LOH colonies remained unchanged, crossovers were sometimes observed (9.5% of LOH colonies) between ChIII and the homologous minichromosome, Ch^16^ (Figure 2A, lane 4). Importantly, minichromosomes from the remaining 90.5% of the LOH colonies appeared truncated, as determined by high-resolution separation of chromosomal DNA by PFGE (Figure 2B). As break-induced LOH retained the ade6 marker ∼25kb centromere proximal to the break site (Figure 1A), this raised the possibility that Ch^16^ truncations resulted from *dn*TA, as was previously observed in a *rad55Δ* background (41). This possibility was examined by colony PCR amplification using primers annealing to *rad21^+^* (centromere proximal to the *MAT*a break site) and a telomere specific primer. A PCR product of 300 bp following digestion with MfeI (a restriction site just upstream of the *MAT*a site) was scored positive for *dn*TA. Sequence analysis of the PCR products indicated the presence of ∼300 bp of G_2–5_TTACA_0–1_ repeats, consistent with *dn*TA at, or very close to, the break site in 13 of the LOH colonies tested (Figure 2C). In 6 of the remaining colonies, telomeres were added ∼9–19 kb centromere proximal to the break site. In full, 24.7% of colonies underwent *dn*TA in *rqh1Δ rad55Δ* strains, which equated to a 1450-fold increase in *dn*TA compared to wild type (0.017%) (Figure 2D, Table 1).

### Suppression of *de novo* telomere addition requires Rqh1 helicase activity

**Figure 3.**
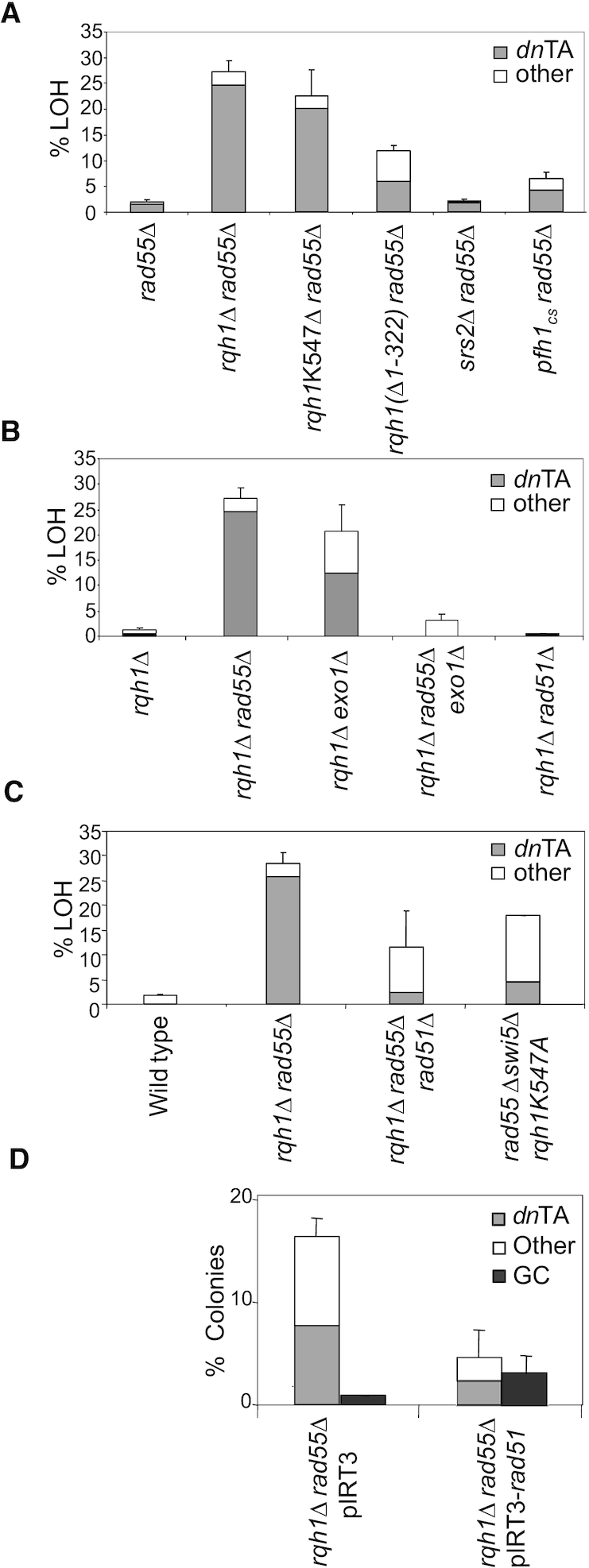
Suppression of LOH by *de novo* telomere addition in *rad55Δ* and *rqh1Δ* mutant backgrounds. (**A**) Mechanisms of LOH observed when *rad55^+^* is deleted in various DNA helicase mutant backgrounds (Table 1). (**B**) Mechanisms of LOH observed when *rqh1^+^* is deleted in various HR mutant backgrounds (Table 1, Supplementary Table SI). (**C**) Effect of abrogation of Rad51 loading on *dn*TA and LOH in an *rqh1Δ* background. (**D**) Graph depicting analysis of *dn*TA and GC levels following over-expression of either an empty vector (pIRT3) or Rad51 (pIRT3-*rad51*) in an *rqh1Δ rad55Δ* background (Table 2).

To determine whether Rqh1 required its helicase activity to suppress *dn*TA, we introduced a helicase-dead mutation *rqh1-K547A* (19,51) into a *rad55Δ* background. In this strain, levels of break-induced LOH and *dn*TA resembled those observed in the *rqh1Δ rad55Δ* strain (Figure 3A, Table 1) suggesting Rqh1 helicase activity is required to suppress *dn*TA in a *rad55Δ* background.

Rqh1 has been shown to function in a complex with Top3 (19,20,52). As the *top3Δ* strain is non-viable (19,52), we tested the requirement of the Top3 interaction in suppressing *dn*TA using an *rqh1ΔN1–322* mutant which has lost the Top3 binding domain (20) and is expressed at similar levels to the wild-type Rqh1 (20). DSB-induced LOH in the *rad55Δrqh1ΔN1–322* mutant was significantly higher (11.9%, *P*<0.001) than that observed in *rad55Δ* (1.8%), but less than the *rqh1Δ rad55Δ* strain (27.3%; Figure 3A; Table 1). This effect could be attributed to a requirement for Rqh1-Top3 interaction in suppressing break-induced LOH in a *rad55Δ* background or to partial loss of Rqh1 helicase activity in the *rqh1ΔN1–322* mutant.

To determine whether other helicases could function similarly to Rqh1, we introduced a deletion of *srs2^+^* or a cold-sensitive allele of *pfh1^+^*, pfh1-R20 (*pfh1_cs_*) (45) into a *rad55Δ* background and examined levels of *dn*TA. Srs2 is implicated in regulation of HR where it antagonizes the activity of the Rad55–Rad57 heterodimer (53–55). In contrast to the *rqh1Δ* mutant, the *srs2Δ* mutant failed to significantly increase levels of break-induced LOH in a *rad55Δ* background (Figure 3A; Table 1). The *S. cerevisiae* Pfh1 homologue, Pif1 has been identified as a suppressor of *dn*TA and gross chromosomal rearrangements (56,57). The *rad55Δ pfh1_cs_* strain showed a modest yet significant increase in LOH (6.4%) compared to wild-type background at semi-permissive temperature (0.8%, *P* = 0.013), and a significant increase in comparison to *rad55Δ* LOH levels (*P* = 0.027), at semi-permissive temperature (Figure 3A; Table 1). Therefore, Pfh1 can suppress LOH arising predominantly from *dn*TA, in accordance with the described role in *S. cerevisiae*. However, in our assay, Rqh1 helicase clearly plays a more prominent role in suppressing *dn*TA than Srs2 or Pfh1.

### Rqh1 functions with early acting HR proteins to suppress *dn*TA

Next we wished to examine the potential role of other HR factors in suppressing *dn*TA in an *rqh1Δ* background. As Exo1 functions early in HR during DSB resection, we examined the relationship between Rqh1 and Exo1 (5,6,58). Deletion of *exo1*^+^ did not significantly alter levels of break-induced LOH compared to wild type (41). However, a striking increase in levels of break-induced LOH (20.7%) was observed in an *rqh1*Δ *exo1*Δ double mutant, 60% (12 of 20 examined colonies) of which was due to *dn*TA (Figure 3B, Table 1). Break-induced marker loss after deletion of *exo1*^+^ in an *rqh1Δ* or *rqh1Δrad55Δ* background was also determined (Table 1). Break-induced LOH was significantly reduced in the *rqh1Δ rad55Δ exo1Δ* mutant compared to the *rqh1Δ rad55Δ* mutant (*P*<0.001) and no *dn*TA products were obtained upon further analysis (Table 1). This requirement for *exo1*^+^ in facilitating *dn*TA in the *rqh1Δ rad55Δ* background is in accordance with Exo1-dependent end-resection facilitating *dn*TA, as previously proposed (41). We were unable to test the role of Rad52 in suppressing *dn*TA as the *rqh1Δrad52Δ* strain was extremely sick, consistent with previously reported findings (19).

As Rad57 forms a heterodimer with Rad55 (59), we examined gene marker loss in a *rad57Δ* background. The resultant marker loss profile was similar to that of *rad55*Δ strains (Table 1). Following DSB induction in an *rqh1Δ rad57Δ* background, 7.7% of colonies exhibited extensive LOH (*P* = 0.011 compared to a *rad57Δ* single mutant), out of which 50% of the double mutant had undergone *dn*TA (Table 1). Thus, Rqh1 can suppress LOH in a *rad57Δ* background, albeit not to the same extent as in a *rad55Δ* background.

DSB induction within Ch^16^ in a *rad51Δ* background has previously been shown to result in a higher proportion of minichromosome loss, demonstrating a failure to repair the DSB (41,42). DSB induction in a *rqh1Δ rad51Δ* background resulted in reduced levels (0.39%) of LOH colonies compared to wild type (1.7%; *P* = 0.007) and *rqh1Δ* (1.5%; *P* = 0.051), indicating that, in contrast to a *rad55Δ* background, Rqh1 does not suppress break-induced LOH significantly in a rad*51*Δ background (Figure 3B; Table 1). Instead, a significant increase in NHEJ/SCR (76.8%) was observed in an *rqh1Δ rad51Δ* background compared to that of *rad51*Δ (35.9%, (41)), indicating that DSBs in an *rqh1*Δ *rad51*Δ double mutant are still competent for HR-independent repair, even though HR is severely impaired. These observations are consistent with an early role for Rqh1 in HR, as described for Sgs1 and BLM in budding yeast and human cells, respectively (4–6,58).

We have previously shown that LOH is significantly reduced in *mus81Δ* (0.2%) compared to *rad55Δ* strain (1.8%, *P* = 0.014) (41). In a *mus81Δ rad55Δ* strain, Ch^16^ loss dramatically increased (60.5%) compared the *mus81Δ* (38.1%) or *rad55Δ* (30.5%) single mutants (41). As expected, GC is dramatically reduced in *mus81Δ rad55Δ* (5.1%) compared to *mus81Δ* (29.0%) as Rad55 acts upstream of Mus81 in HR. Consistent with the late role of Mus81 in HR, SCR in *mus81Δ rad55Δ* (23.4%) is similar to *mus81Δ* (28.1%) in comparison to *rad55Δ* (62.8%) (41). Although LOH was not measured in *mus81Δ rad55Δ*, the high levels of Ch^16^ loss in *mus81Δ rad55Δ* and the low levels of LOH in *mus81Δ* suggest that Mus81 does not suppress *dn*TA in an *rhp55Δ* background.

### A critical role for Rad51 loading in facilitating efficient *dn*TA

Following DSB induction, *dn*TA may occur before or after Rad51-dependent strand invasion. If *dn*TA resulted immediately following resection, then this event should be Rad51-independent. In an *rqh1Δ rad55Δ rad51*Δ triple mutant, increased levels of LOH (11.7%) were observed, although only 21.1% of these resulted from *dn*TA (Figure 3C; Table 2). Thus, *dn*TA in an *rqh1Δ rad55Δ rad51*Δ background was reduced almost ten-fold from 24.7% to 2.5% compared to the *rqh1Δ rad55Δ* background (Tables 1 and 2). Thus Rad51 contributes to *dn*TA in an *rqh1Δ rad55Δ* background.

**Table 2.** Rad51 loading promotes *de novo* telomere addition in *rqh1D rad55D*

Ch16-MGH in genetic background	% ade^+^ G418^S^/Hyg^S^ his^+^ (GC)	% ade^+^ G418^R^/Hyg ^R^ his^+^ (NHEJ/SCR)	% ade^−^ G418^S^/Hyg^S^ his^−^ (Ch^16^ loss)	% ade^+^ G418^S^/Hyg^S^ his^−^ (LOH)	% ade^+^ G418^S^/Hyg^S^ his^−^ (*dn*TA)	*P value* (LOH relative to wildtype)
*Wild type*	49.7 ± 2.6	25.0 ± 1.4	20.5 ± 2.6	1.7 ± 0.3	0.0% (0/20)	1.000
*rqh1Δ rad55Δ rad51Δ*	0.1 ± 0.06	67.0 ± 12.7	21.0 ± 6.3	11.7 ± 7.2	2.5% (4/20)	0.161
*swi5Δ*	21.9 ± 2.6	57.2 ± 3.2	20.5 ± 2.6	0.8 ± 0.1	0.3% (7/20)	0.023
*rad55Δ swi5Δ*	0.14 ± 0.07	30.2 ± 0.5	65.9 ± 0.3	3.6 ± 0.9	1.9% (10/20)	0.435
*rqh1*K547A *swi5Δ*	15.4 ± 3.3	60.5 ± 2.4	13.2 ± 4.8	5.6 ± 0.4	2.3% (8/20)	0.028
*rqh1*K547A *rad55Δ swi5Δ*	0.15 ± 0.15	36.9 ± 1.5	44.9 ± 0.3	17.9 ± 0.01	4.5% (5/20)	3.28E–07
*rad55Δ rqh1Δ+* pIRT3	0.7 ± 0.07	68.4 ± 3.1	14.4 ± 2.6	16.4 ± 1.5	9.8% (12/20)	1.27E–05
*rad55Δ rqh1Δ+* pIRT3*-rad51*	3.3 ± 1.7	88.3 ± 5.9	3.9 ± 2.0	4.5 ± 2.5	2.3% (10/20)	0.005

The mean ± SE from at least three independent experiments with three individual strains are shown. % *dn*TA was calculated by multiplying the fraction of *dn*TA positive colonies identified from the 20 ade+ G418S/HygS colonies examined (indicated in brackets) by the % LOH. The *P*-value for *rad55∆ rqh1∆+* pIRT3-rad51 is 0.042 relative to *rad55∆ rqh1∆+* pIRT3. Wt values presented as previously described (Cullen *et al.*, 2007)

The Swi5/Sfr1 heterodimer functions in parallel to the Rad55/Rad57 heterodimer to load Rad51. DSB induction in a *swi5Δ* background resulted in significantly increased levels of NHEJ/SCR colonies (57%, *P* < 0.001) compared to wild type (25%), resembling levels observed in a *rad55Δ* background (63%). GC (22% *P* < 0.001) was significantly reduced in a *swi5Δ* background compared to wild type (50%), but levels of Ch16 loss (20.5%) and LOH (0.8%) were similar to wild type (Table 2).

We also tested the *rad55Δ swi5Δ* double mutant. Marker loss in a *rad55Δ swi5Δ* strain was very similar to that in a *rad51*Δ strain, resulting in high levels of Ch^16^ loss (66%), consistent with failed Rad51 loading. Levels of NHEJ/SCR colonies were also reduced in the *rad55Δ swi5Δ* background (30%) compared to *rad55Δ* (62%) or *swi5Δ* (57%) single mutants, consistent with this population arising through HR-dependent SCR. Further, levels of LOH through *dn*TA (1.9%) in *rad55Δ swi5Δ* strain were equivalent to that of *rad55Δ* strain alone (1.4%: *P* = 0.743: Table 2).

Introducing a helicase-dead *rqh1* mutant into a *rad55Δ swi5*Δ background (*rad55Δ swi5Δ rqh1-K547A*) resulted in a striking increase in break-induced LOH (17.9%, *P*<0.001) compared to wild type. However, further analysis indicated that only 25% of these were a result of *dn*TA (4.5%; Figure 3C; Table 2). Interestingly, *dn*TA levels were reduced 4.5-fold in *rqh1-K547A rad55Δ swi5Δ* background compared to an *rqh1-K547A rad55Δ* background (20.5%; Table 1). Therefore efficient *dn*TA in *rad55Δ* strains in the absence of Rqh1 helicase activity requires Swi5 or Rad51, thus further indicating a role for Rad51-loading being required for efficient *dn*TA.

We have previously demonstrated that Rad51 overexpression (*OPrad51*) reduced levels of *dn*TA in a *rad55Δ* background (41), consistent with competition between the Rad51 recombinase and telomerase for resected ends. We therefore tested whether Rad51 overexpression could similarly reduce levels of *dn*TA observed in an *rqh1Δ rad55Δ* background by introducing pIRT3-*rad51* (60). *OPrad51* resulted in significantly increased levels of GC (3.3%, *P* = 0.05), and SCR (88.3%, *P* = 0.03), and significantly reduced levels of Ch16 loss (3.9%, *P* = 0.04) and LOH (4.45%, *P* = 0.04), and therefore reduced levels of *dn*TA, in an *rqh1Δ rad55Δ* background compared to vector alone (Figure 3D; Table 2). However, whilst *rad55Δ*, *rqh1Δ* and *rad55Δ rqh1Δ* are exquisitely sensitive to radiation in a rad51Δ background (Supplementary Figure S2), overexpression of Rad51 does not significantly rescue radiation sensitivity in these mutants (Supplementary Figure S3). Together, these data identify a critical role for Rad51 recombinase levels in facilitating *dn*TA in an *rqh1Δ rad55Δ* background.

### The MRN complex promotes SCR and partially suppresses *dn*TA in a *rad55Δ* background

We wished to examine whether deletion of other early acting HR genes could suppress *dn*TA in a *rad55Δ* background. We previously reported that deleting *exo1^+^* in a *rad55Δ* background resulted in reduced levels of *dn*TA compared to *rad55Δ* strains (41). Here we further tested the effect of deleting the MRN complex in a *rad55Δ* background. Deletion of *mre11^+^, rad50^+^* or *nbs1^+^* in a *rad55Δ* background resulted in a striking reduction in levels of NHEJ/SCR colonies and increased levels of Ch^16^ loss compared to *rad55Δ* (Table 3). These results resemble those of another HR mutant, *rad55Δ rad51Δ* (42), and are consistent with the break-induced ade^+^ G418^R^ his^+^ population in a *rad55Δ* background resulting from HR-dependent SCR following break-induction. Levels of LOH colonies arising through *dn*TA were modestly increased in *rad55Δ mre11Δ* (4.8%), *rad55Δ rad50Δ* (4.5%), and *rad55Δ nbs1Δ* (2.4%) compared to *rad55Δ* (1.4%) or the respective individual MRN deletion mutants (Table 3). Thus, while the MRN complex is important for SCR, it performs only a minor role in suppressing *dn*TA in a *rad55Δ* background compared to Rqh1. Thus, Rqh1 plays a functionally distinct role from Exo1 and the MRN complex in suppressing *dn*TA in *rad55Δ* strains.

**Table 3.** DSB-induced marker loss and *dn*TA in *rad55Δ* and MRN deletion mutants

Ch16-MGH in genetic background	% ade^+^ G418^S^/Hyg^S^ his^+^ (GC)	% ade^+^ G418^R^/Hyg ^R^ his^+^ (NHEJ/SCR)	% ade^−^ G418^S^/Hyg^S^ his^−^ (Ch^16^ loss)	% ade^+^ G418^S^/Hyg^S^ his^−^ (LOH)	% ade^+^ G418^S^/Hyg^S^ his^−^ (*dn*TA)	*P value* (LOH relative to wildtype)
*Wild type*	49.7 ± 2.6	25.0 ± 1.4	20.5 ± 2.6	1.7 ± 0.3	0.0% (0/20)	1.000
rad55Δ*	2.9 ± 0.7	62.8 ± 9.8	30.5 ± 10.9	1.8 ± 1.1	1.4% (16/20)	0.936
mre11Δ*	30.7 ± 2.0	25.6 ± 5.1	35.7 ± 5.8	0.6 ± 0.2	0.3% (11/21)	0.013
*mre11Δ rad55Δ*	0.6 ± 0.4	20.1 ± 0.2	61.7 ± 0.1	6.6 ± 0.2	4.8% (16/22)	<0.05
rad50Δ*	18.3 ± 1.8	23.9 ± 0.6	49.7 ± 1.9	0.6 ± 0.2	0.2% (6/20)	0.011
*rad50Δ rad55Δ*	1.0 ± 0.3	29.2 ± 6.3	53.9 ± 5.4	4.9 ± 0.7	4.5% (21/23)	0.088
nbs1Δ *	15.6 ± 0.7	30.9 ± 2.6	43.6 ± 3.3	1.4 ± 0.2	1.7% (17/21)	0.441
*nbs1Δ rad55Δ*	0.1 ± 0.1	23.4 ± 1.9	61.9 ± 1.6	2.6 ± 0.5	2.4% (20/22)	0.163

The mean ± SE from at least three independent experiments with three individual strains are shown. % *dn*TA was calculated by multiplying the fraction of *dn*TA positive colonies identified from the 20 ade+ G418S/HygS colonies examined (indicated in brackets) by the % LOH. * denotes values as previously described, shown here for comparison (Cullen *et al.*, 2007)

### Striking levels of *dn*TA at a DSB lacking a homologous distal chromosome arm

**Figure 4.**
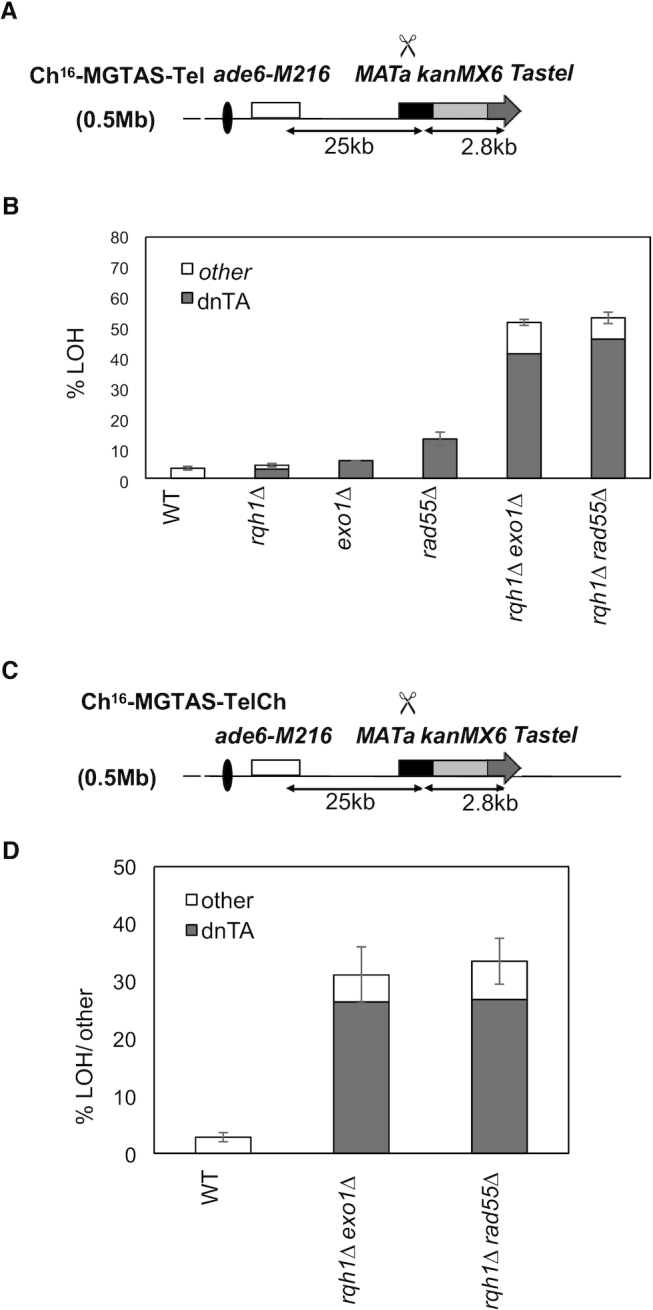
Efficient *dn*TA occurs at a DSB lacking a homologous distal chromosome arm. (**A**) Schematic of the Ch^16^-MGTASTel minichromosome. ChIII as described in Figure 1B. The loci of the centromeres (black oval), *ade6-M216* and *ade6-M210* alleles (white boxes), *MAT*a target site (black box), *KanMX6* gene (gray), and *TASTel* sequence (grey arrow) as indicated. pREP81X-HO generates a DSB at the *MAT*a target site (scissors). (**B**) Histogram of percentage break-induced LOH arising through *dn*TA (grey) or other (white) in wild type (WT, TH2039), *rqh1Δ* (TH2254), *exo1Δ* (TH2420), *rad55Δ (*TH2253), *rqh1Δ exo1Δ* (TH8226) and *rqh1Δ rad55Δ* (TH2266) strains following HO-endonuclease induction for 48h (Table 1). (**C**) Schematic of the Ch^16^-MGTASTelCh minichromosome. Minichromosome whose features are described in (A); ChIII as described in Figure 1B. (**D**) Histogram of percentage break-induced LOH arising through *dn*TA (gray) or other (white) in wild-type (TH8597), *rqh1Δ exo1Δ* double mutant (TH8598) and *rqh1Δ rad55Δ* double mutant (TH8708) strains following HO-endonuclease induction for 48 h (Table 5).

The above findings indicate that efficient *dn*TA is associated with HR intermediates. To test this further, we asked whether *dn*TA would be further increased under circumstances in which post-synaptic second-end capture was abrogated. To address this, the (130 kb) homologous arm centromere-distal to the break site was replaced by a construct containing the 1.8 kb *MATa* target sequence/G418-resistant marker and a 1 kb synthetic telomere, TASTel fragment containing 700 bp of subtelomeric DNA (TAS) and 300 bp of telomeric DNA (Tel) (Figure 4A) (61), in which there is no distal homologous chromosome arm (Ch^16^-MGTASTel). Following break-induction, DSB repair by NHEJ/SSC in Ch^16^-MGTASTel results in cells that retain the ade^+^ G418^R^ phenotype. Cells that fail to repair the DSB lose the minichromosome and become ade^−^ G418^S^, while those which undergo LOH become ade^+^ G418^S^. Following DSB induction in Ch^16^-MGTASTel cells, homology search, strand invasion and DNA synthesis steps should still be possible for the broken centromere-proximal arm, while the later HR stages of second-end capture or strand annealing are obviated. In contrast to DSB induction in the Ch^16^-MGH strain, it is not possible for Ch^16^-MGTASTel cells to undergo GC and become ade^+^ G418^S^ since GC requires the participation of two homologous DSB arms (62).

We found DSB induction in Ch^16^-MGTASTel in a wild-type background resulted in 79.3% of the colonies becoming ade^−^ G418^S^, consistent with very high levels of unrepaired breaks leading to chromosome loss or other undetectable rearrangements; 17.4% remained ade^+^ G418^R^, consistent with NHEJ or SCR; and 3.3% became ade^+^ G418^S^, having undergone LOH (Table 4). Further PCR analysis of 20 individually isolated ade^+^ G418^S^ colonies failed to detect *dnTA* (Figure 4B; Table 4). Deletion of *rqh1*^+^, *exo1*^+^ or *rad55^+^* each resulted in increased levels of NHEJ/SCR and reduced Ch^16^ loss, as was observed in Ch^16^-MGH. This was associated with modest increases in LOH and *dn*TA with 13% *dn*TA noted in a *rad55Δ* background (Figure 4B; Table 4). Remarkably, DSB induction in an *rqh1Δ rad55Δ* background resulted in 53% of the colonies becoming ade^+^ G418^S^, corresponding to 45.2% *dnTA* (Figure 4B; Table 4). Similarly, following DSB induction in an *rqh1Δ exo1Δ* background, 51% of the colonies became ade^+^ G418^S^ which corresponded to 41.4% *dn*TA.

**Table 4.** DSB-induced marker loss and *dn*TA in minichromosome

Ch^16^-MGTASTel					
Ch16-MGTASTel genetic background (strain number)	% ade^+^ G418^R^ (NHEJ/ SCR/ uncut)	% ade^−^ G418^S^ (Ch^16^/loss/ other)	% ade^+^ G418^S^ (LOH)	% ade^+^ G418^S^ (*dn*TA)	*P-value* (LOH relative to wild type)
*Wild type*	17.4 ± 4.0	79.3 ± 4.17	3.3 ± 0.6	0.0% (0/20)	1.000
*rqh1Δ*	50.8 ± 0.4	44.8 ± 0.8	4.3 ± 0.6	3.0% (14/20)	<0.005
*exo1Δ*	51.0 ± 0.8	43.2 ± 0.7	5.8 ± 0.1	5.8% (20/20)	<0.005
*rad55Δ*	47.2 ± 3.9	39.7 ± 1.8	13.0 ± 2.3	13% (20/20)	<0.005
*rqh1Δ exo1Δ*	23.3 ± 2.9	25.0 ± 4.5	51.7 ± 1	41.4% (16/20)	<0.005
*rqh1Δ rad55Δ*	31.3 ± 0.8	15.5 ± 1.1	53.2 ± 1.9	45.2% (17/20)	<0.005

The mean **±** SE from at least three independent experiments with three individual strains are shown. % *dn*TA was calculated by multiplying the fraction of *dn*TA positive colonies identified from the 20 ade+ G418S colonies examined (indicated in brackets) by the % LOH.

To test whether the increased levels of *dn*TA resulted from loss of the second homologous chromosome arm, or from proximity to the TASTel synthetic telomere sequence, an additional minichromosome was constructed in which the TASTel sequence was integrated distal to the *MAT*a site of Ch^16^-MG, (in the same locus as Ch^16^-MGH), but retaining the distal arm of the minichromosome, to form Ch^16^-MG(TASTel)Ch (Figure 4C). Surprisingly, DSB induction in a wild-type strain containing Ch^16^-MG(TASTel)Ch resulted in 76% Ch^16^ loss or extensive LOH; while 2% of the colonies underwent LOH or GC, and *dn*TA was not detected (Table 5). Although we cannot distinguish LOH or GC colonies, the levels of ade^+^ G418^S^ colonies (combining LOH and GC) in Ch^16^-MG(TASTel)Ch were much less than ade^+^ G418^S^ his^−^ (GC) in Ch^16^-MGH cells. The reduced GC observed in Ch^16^-MG(TASTel)Ch strain presumably reflects the reduced homology with ChIII due to the addition of the non-homologous TASTel cassette. Following DSB induction in an *rqh1Δ rad55Δ* background, 33% of colonies were ade^+^ G418^S^, corresponding to 27% *dn*TA (Figure 4D; Table 5). This was again significantly reduced compared to 45.2% *dn*TA (*P* = 0.0142) observed using Ch^16^-MGTASTel, but was very similar to levels observed using Ch^16^-MGH (25%). Following DSB induction within an *rqh1Δ exo1Δ* background, in which GC was abrogated, 31% of the colonies were ade^+^ G418^S^, corresponding to 26% *dn*TA (Figure 4D; Table 5). This was significantly reduced compared to that observed using Ch^16^-MGTASTel (41.4% *P* = 0.04), but was greater than levels observed using Ch^16^-MGH (12% *P*>0.05). Thus, *dn*TA was significantly further increased in the context of a ‘one-armed’ break in either *rqh1Δ rad55Δ* or *rqh1Δ exo1Δ* backgrounds. While in the *rqh1Δ exo1Δ* background, proximity of the DSB site to telomeric sequence could contribute to high *dn*TA levels, the striking *dn*TA levels observed in an *rqh1Δ rad55Δ* background are consistent with disrupting post-synaptic HR events.

**Table 5.** Marker loss and *dn*TA in minichromosome Ch^16^-MG(TASTel)Ch

Ch^16^-MG(TASTel)Ch genetic background (strain number)	% ade^+^ G418^R^ (NHEJ/ SCR/ uncut)	% ade^−^ G418^S^ (LOH/ Ch^16^ loss)	% ade^+^ G418^S^ (LOH/GC)	% ade^+^ G418^S^ (*dn*TA)	*P-value* (LOH relative to wild type)
*Wild type*	21.2 ± 5.6	76± 6.0	2.8 ± 0.8	0.0% (0/20)	1.000
*rqh1Δ exoΔ*	28.3 ± 5.6	40.6 ± 3.6	31.1 ± 4.9	26.4% (17/20)	0.0072
*rqh1Δ rad55Δ*	25.5 ± 1.0	41.0 ± 2.5	33.5± 4.0	26.8% (16/20)	0.0058

The mean **±** SE from at least three independent experiments with three individual strains are shown. % *dn*TA was calculated by multiplying the fraction of *dn*TA positive colonies identified from the 20 ade+ G418S colonies examined (indicated in brackets) by the % LOH.

## DISCUSSION

Here we describe roles for the BLM homologue, Rqh1 helicase and the Rad51 paralog, Rad55, in both facilitating homologous recombination and in suppressing chromosome healing at a break site in fission yeast. We find Rqh1 helicase, together with either Rad55 or Exo1 suppresses *dn*TA. Further, we find that a DSB lacking a homologous distal chromosome arm undergoes highly efficient *dn*TA in these genetic backgrounds. Together these findings indicate that chromosome healing can occur highly efficiently within HR intermediates. Here we consider the mechanisms by which these events occur in the absence of these genes and the implications for genome stability.

### Suppressing chromosome healing

**Figure 5.**
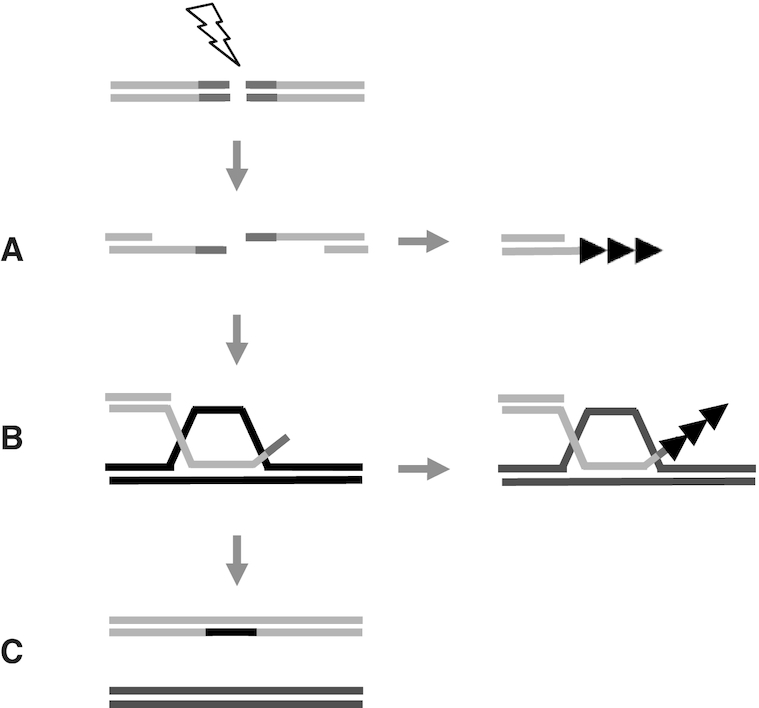
Model for efficient *dn*TA within HR intermediates. (**A**) Presynaptic break-induced *dn*TA in an *rqh1Δ rad55Δ* background. Following DSB induction at the *MAT*a site (dark grey) within the minichromosome (light grey) reduced resection, shortened and or an altered Rad51 nucleofilament structure facilitates presynaptic *dn*TA (black arrows). (**B**) Postsynaptic break-induced *dn*TA in an *rqh1Δ rad55Δ* background. Following DSB induction Rad51-dependent strand invasion of ChIII (black) leads to D-loop formation, which is stabilized in the absence of both Rqh1, and Rad55. Non-homologous *MAT*a 3′ ends remain unprocessed and are extruded from the D-loop, facilitating *dn*TA. Removal of the second homologous arm significantly further increases *dn*TA in this context suggesting that second end capture or strand annealing efficiently suppresses *dn*TA. (**C**) Over-expression of Rad51 in an *rqh1Δ rad55Δ* background increases gene conversion. Thus Rad51 loading and subsequent nucleofilament structure plays a critical role in defining the fate of broken chromosome ends. Pathways A and B may be non-exclusive. See text for details.

Our study identifies an independent role for the HR proteins Rqh1, together with Rad55 or Exo1 in suppressing *dn*TA, with a striking increase in *dn*TA being observed in *rqh1Δ rad55Δ* and to a lesser extent *rqh1Δ exo1Δ* backgrounds, compared to wild type. As extensive resection requires both Rqh1 and Exo1 (4–6), these findings are consistent with partially resected ends acting as efficient substrates for *dn*TA (37,38). Loss of both Rad55 and Rqh1 may also facilitate presynaptic *dn*TA either through reduced resection or through altering the structure of the Rad51 nucleofilament so that it is more conducive to *dn*TA (Figure 5) (63–65). That overexpression of Rad51 in an *rqh1Δ rad55Δ* background led to significantly reduced levels of *dn*TA and significantly elevated levels of both GC and SCR suggests that Rad55 suppresses *dn*TA through facilitating efficient Rad51 assembly. These findings are broadly consistent with a role for HR in preventing *dn*TA through competition for resected ends (41).

However, RecQ helicase activity is also required for branch migration, to displace non-allelic recombination, and to prevent the formation of double-Holliday junctions (15), and loss of these post-synaptic functions may also facilitate *dn*TA. Here, loss of Rqh1 helicase activity may lead to stabilization of the invading strand in a D-loop, which may facilitate *dn*TA (Figure 5). Consistent with this, *dn*TA was occasionally associated with crossover events between Ch^16^ and ChIII in *rqh1Δ rad55Δ* (Figure 3A). Loss of Rad55 may also contribute to post-synaptic *dn*TA by promoting Srs2-dependent exclusion of the non-homologous MATa site from the D-loop, which may now act as a landing pad for telomerase. Accordingly, postsynaptic roles have also been assigned to the Rad51 paralogues, which are the human counterparts to Rad55 and Rad57 (66,67). In this respect, the Rad51L3–XRCC2 complex physically interacts with and stimulates BLM to disrupt Holliday junctions *in vitro* (67). The observation that a DSB lacking a homologous distal chromosome arm significantly further increased *dn*TA levels in *rqh1Δ rad55Δ* or *rqh1Δ exo1Δ* backgrounds is consistent with a model in which the post-synaptic HR events of second end capture or strand annealing compete with dnTA (Figure 5).

### Determinants of chromosome healing

We found Rad51 to be required for efficient *dn*TA. This was unexpected as efficient Rad51 loading suppresses *dn*TA. Accordingly, *dn*TA levels were significantly reduced in a *rad51*Δ *rqh1*Δ *rad55Δ* strain compared to an *rqh1Δ rad55Δ* background. Similarly, preventing Rad51 loading by simultaneously disrupting both Rad55–Rad57 and Swi5–Sfr1 heterodimers significantly reduced *dn*TA in *swi5Δ rad55Δ rqh1-K547A* compared to a *rad55Δ rqh1-K547A* background. Taken together, our data are consistent with the hypothesis that Rad55–Rad57 and Swi5–Sfr1 have distinct roles in Rad51 assembly (68). It has been shown that Rad51-foci form less efficiently in Swi5–Sfr1 compared to a Rad55–Rad57 mutant (11) whereas Rad55–Rad57 subtly organizes the Rad51-nucleofilament structure (55). Therefore, Swi5–Srf1 is required to stabilize Rad51, thus promoting *dn*TA, whereas Rad55–Rad57 is required to modulate Rad51 structure, which promotes GC and suppresses *dn*TA.

As Rad51 over expression suppressed dnTA and promoted GC in an *rqh1Δ rad55Δ* background, this suggests the Rad51 nucleofilament structure is likely to play a critical role in defining the fate of broken chromosome ends. Rad51 may potentially promote *dn*TA presynaptically in this respect, through assembling a nucleofilament structure to which telomerase may preferentially bind in the absence of Rad55 and Rqh1. Rad51 may also promote *dn*TA post-synaptically, through facilitating D-loop formation, which in the absence of Rqh1 or Rad55 facilitates dnTA at non-homologous ends extruded from the D-loop (Figure 5). As D-loops are structurally analogous to T-loops (69), our findings suggest a structural context through which T-loops may promote telomerase activity.

We found Exo1 to be required for *dn*TA in an *rqh1Δ rad55Δ* background. As efficient *dn*TA was observed in an *rqh1Δ exo1Δ* background this indicates that Exo1 is not required for telomere addition. However, in *S. cerevisiae* Sae2/MRX and Sgs1 activities are required to allow Exo1 access, which contributes to telomere end processing and elongation (70). We speculate that further loss of Exo1-dependent resection in an *rqh1Δ rad55Δ* background fails to generate sufficient ssDNA necessary to facilitate *dn*TA. Such resection may facilitate Rad51 binding as indicated above. Reduced *dn*TA is associated with increased NHEJ/SCR following Exo1 deletion in an *rqh1Δ rad55Δ* background. However, further studies will be required to elucidate the precise role of Exo1 in this context.

### Mechanisms of telomere addition

The finding that efficient *dn*TA was observed at or near the *MATa* site was unexpected, as this region lacks the canonical GGTTACA *S. pombe* telomeric repeat sequence (61). Studies in *S. cerevisiae* have shown *dn*TA was restricted to very short regions of homology to the telomerase guide RNA that were likely to facilitate annealing of such RNA (71,72). Thus, efficient *dn*TA observed in our study may result from recognition of degenerate telomeric sequences by guide RNA or other telomere recruitment factors. Alternatively, telomere recruitment may be achieved in a sequence-independent manner through interaction between ssDNA binding factors and telomerase (73).

### Chromosome healing and genome instability

Our findings indicate that *dnTA* has the capacity to stabilize broken chromosomes. However, such a role comes at the price of potentially extensive loss of genetic material centromere-distal to the break site. While *dn*TA is predicted to result in loss of viability in a haploid setting, such extensive LOH in a diploid or polyploidy cells may be tolerated. Thus, *dn*TA may provide an important back-up mechanism to rescue broken chromosomes, thus facilitating cell survival.

## Supplementary Material

gkz1109_Supplemental_File
