# Supplementary material for: Homologous recombination repair intermediates promote efficient de novo telomere addition at DNA double-strand breaks
Source: Nucleic Acids Res. 2019 Dec 12;48(3):1271–84. doi: 10.1093/nar/gkz1109 (PMC7026635; doi:10.1093/nar/gkz1109)
Supplement: gkz1109_Supplemental_File [file gkz1109_supplemental_file.docx]

**Supplementary Data**

**Homologous recombination repair intermediates promote efficient *de novo* telomere addition at DNA double-strand breaks**

Hulme *et al.*

Table S1

Figure S1

Figure S2

Figure S3

**Figure S1:** Spot dilution assays of *wild-type* *rad55∆, rqh1∆* and *rqh1∆ rad55∆* strains containing Ch^16^-MGH and transformed with *rep81X*-HO plasmid following DSB induction in the absence of thiamine. Cultures of the above strains were grown for 24 h with or without thiamine before washing, diluting and spotting onto plates with or without thiamine, as indicated.

**Figure S2:** Radiation sensitivity of *rad55Δ, rqh1Δ,* and *rad55Δrqh1Δ* mutants in the context of *rad51Δ.* Cultures of the above strains were grown for 24 h in YE6S to mid-log phase (0.2 OD). 100-fold dilutions were exposed to 0 Gy or 100 Gy and plated on YE6S to form colonies. Three biological repeats were performed for each strain, each with three technical repeats, and error bars of standard deviation are shown.

**Figure S3:** Radiation sensitivity of *rad55Δ, rqh1Δ,* and *rad55Δrqh1Δ* mutants in the context of the overexpression of Rad51^+^ overexpression. Cultures of the above strains were grown for 24 h in EMM-leucine to mid-log phase (0.2 OD). 100-fold dilutions were exposed to 0 Gy or 100 Gy and plated on YE6S to form colonies. Three biological repeats were performed for each strain, each with three technical repeats, and error bars of standard deviation are shown.

**Table S1: Strains used in this study**

| **Strain Number** | **Genotype** |
| --- | --- |
| **TH1230** | *leu1-32, ade6-M210, ura4-D18, his3-D1,* Ch16-MGH, (Ch16 ade6-M216 rad21::MATa kanMX6 cid2::his3+), *h^-^* |
| **TH1527** | *rad51::ura4^+^*, *leu1-32, ade6-M210, ura4-D18, his3-D1,* *h^+^,* Ch16-MGH |
| **TH1579** | *rad51::ura4^+^*, *leu1-32, ade6-M210, ura4-D18, his3-D1,* *h^+^,* Ch16-MGH, *pREP81X-HO* |
| **TH1588** | *exo1::ura4^+^,* Ch16-MGH, *ura4-D18, leu1-32, ade6-210, his3-D1, h^+^* |
| **TH1607** | *exo1::ura4^+^*, Ch16-MGH, *leu1-32, ade6-M210, ura4-D18, his3-D1,* *h^-^*, *pREP81X-HO* |
| **TH1696** | *srs2::ura4^+^, leu1-32, ade6-M210, ura4-D18, his3-D1,* Ch16-MGH, h+ |
| **TH1711** | *srs2::ura4^+^, leu1-32, ade6-M210, ura4-D18, his3-D1,* Ch16-MGH, h+, *pREP81X-HO* |
| **TH1747** | *rad55::ura4^+^,* *leu1-32, ade6-M210, ura4-D18, his3-D1,* Ch16-MGH  *h+* |
| **TH1760** | *rad55::ura4^+^,* Ch16-MGH, *leu1-32, ade6-M210, ura4-D18, his3-D1,* *h^+^*, *pREP81X-HO* |
| **TH1788** | *rqh1::ura4^+^, leu1-32, ade6-M210, ura4-D18, his3-D1,* *h^+^*, Ch16-MGH, |
| **TH1807** | *rqh1::ura4^+^, leu1-32, ade6-M210, ura4-D18, his3-D1,* *h^+^*, Ch16-MGH, *pREP81X-HO* |
| **TH1873** | *nbs1::ura4^+^, leu1-32, ade6-M210, ura4-D18, his3-D1,* *h^+^*, Ch16-MGH |
| **TH1900** | *leu1-32, ade6-M210, ura4-D18, his3-D1,* Ch16-MGH, *h^+^*, *pREP81X-HO* |
| **TH1934** | *nbs1::ura4^+^, leu1-32, ade6-M210, ura4-D18, his3-D1,* *h^+^*, Ch16-MGH, *pREP81X-HO* |
| **TH2006** | *rad50::kanMX6, leu1-32, ade6-M210, ura4-D18, his3-D1,* *h^+^*, Ch16-MHH |
| **TH2013** | *rad50::kanMX6, leu1-32, ade6-M210, ura4-D18, his3-D1,* *h^+^*, Ch16-MHH, *pREP81X-HO* |
| **TH2039** | *ura4-D18, leu1-32, ade6-M210,* Ch16-MGTASTel, (Ch16 *ade6-M216 spcc132.03::MATa kanMX6* TASTel*)* *h^-^* |
| **TH2121** | *rhp55::ura4^+^, rqh1::ura4^+^, ade6-m210, ura4-D18, leu1-32, his3-D1,* Ch16 MGH, *h^-^* |
| **TH2136** | *rad55::ura4^+^, rqh1::ura4^+^, leu1-32, ade6-M210, ura4-D18, his3-D1,* Ch16-MGH, *h^-^*, *pREP81X-HO* |
| **TH2175** | *rad55::ura4^+^, srs2::ura4^+^, leu1-32, ade6-M210, ura4-D18, his3-D1,* Ch16-MGH, *h^+^* |
| **TH2209** | *rad55::ura4^+^, srs2::ura4^+^, leu1-32, ade6-M210, ura4-D18, his3-D1,* Ch16-MGH, *h^+^*, *pREP81X-HO* |
| **TH2253** | *rad55::ura4^+^, ura4-D18, leu1-32, ade6-M210,* Ch16-MGTASTel |
| **TH2254** | *rqh1::ura4^+^, ura4-D18, leu1-32, ade6-M210,* Ch16-MGTASTel, *h^-^* |
| **TH2266** | *rqh1::ura4^+^, rad55::ura4^+^*^,^ *ura4-D18, leu1-32, ade6-M210,* Ch16-MGTASTel |
| **TH2285** | *rad32::kanMX6, leu1-32, ade6-M210, ura4-D18, his3-D1,* *h^+^*, Ch16-MHH |
| **TH2297** | *rad32::kanMX6, leu1-32, ade6-M210, ura4-D18, his3-D1,* *h^+^*, Ch16-MHH, *pREP81X-HO* |
| **TH2302** | *rad55::ura4^+^, rqh1::rqh1K547A, leu1-32, ade6-M210, ura4-D18, his3-D1* Ch16-MGH, *h^+^* |
| **TH2360** | *rad55::ura4^+^, rqh1::rqh1K547A, leu1-32, ade6-M210, ura4-D18, his3-D1* Ch16-MGH, *h^+^*, *pREP81X-HO* |
| **TH2388** | *rad51::ura4^+^*, *rqh1::ura4^+^, leu1-32, ade6-M210, ura4-D18, his3-D1,* Ch16-MGH, *h^+^* |
| **TH2412** | *ura4-D18, leu1-32, ade6-M210,* Ch16-MGTASTel, *h^-^, pREP81X-HO* |
| **TH2419** | *rad55::ura4^+^, ura4-D18, leu1-32, ade6-M210,* Ch16-MGTASTel*, pREP81X-HO* |
| **TH2420** | *exo1::ura4^+^*, *ura4-D18, leu1-32, ade6-M210,* Ch16-MGTASTel, *h^-^* |
| **TH2421** | *exo1::ura4^+^*, *ura4-D18, leu1-32, ade6-M210,* Ch16-MGTASTel, *h^-^, pREP81X-HO* |
| **TH2424** | *rqh1::ura4^+^, ura4-D18, leu1-32, ade6-M210,* Ch16-MGTASTel, *h^-^, pREP81X-HO* |
| **TH2433** | *rqh1::ura4^+^, rad55::ura4^+^*, *ura4-D18, leu1-32, ade6-M210,* Ch16-MGTASTel, *pREP81X-HO* |
| **TH2503** | *rad57::ura4^+^, rqh1::ura4^+^_,_ leu1-32, ade6-M210, ura4-D18, his3-D1,* Ch16-MGH, *h^-^* |
| **TH2509** | *rad57::ura4^+^, rqh1::ura4^+^_,_ leu1-32, ade6-M210, ura4-D18, his3-D1,* Ch16-MGH, *h^-^*, *pREP81X-HO* |
| **TH2520** | *rad51::ura4^+^*, *rqh1::ura4^+^, leu1-32, ade6-M210, ura4-D18, his3-D1,* Ch16-MGH, *h^+^, pREP81X-HO* |
| **TH2600** | *rhp55::ura4+, rad50::kanMX6, leu1-32, ade6-M210, ura4-D18, his3-D1,* *h^+^*, Ch16-MHH |
| **TH2610** | *rad57::ura4^+^, leu1-32, ade6-M210, ura4-D18, his3-D1,* Ch16-MGH, *h^-^* |
| **TH2672** | *rhp55::ura4+, rad50::kanMX6, leu1-32, ade6-M210, ura4-D18, his3-D1,* *h^+^*, Ch16-MHH, *pREP81X-HO* |
| **TH2716** | *rhp55::ura4+, rad32::kanMX6, leu1-32, ade6-M210, ura4-D18, his3-D1,* *h^+^*, Ch16-MHH |
| **TH2810** | *rhp55::ura4+, rad32::kanMX6, leu1-32, ade6-M210, ura4-D18, his3-D1,* *h^+^*, Ch16-MHH, *pREP81X-HO* |
| **TH2852** | *rad57::ura4^+^, leu1-32, ade6-M210, ura4-D18, his3-D1,* Ch16-MGH, *h^-^*, *pREP81X-HO* |
| **TH2857** | *rad51::ura4^+^*, *rqh1::ura4^+^, leu1-32, ade6-M210, ura4-D18, his3-D1,* Ch16-MGH, *h^+^* |
| **TH2872** | *rad55::arg3^+^*, *rqh1::ura4^+^, leu1-32, ade6-M210, ura4-D18, his3-D1,* arg3-D4, Ch16-MGH, *h^+^* |
| **TH2875** | *rad55::arg3^+^*, *nbs1::ura4^+^, leu1-32, ade6-M210, ura4-D18, his3-D1,* arg3-D4, Ch16-MGH, *h+* |
| **TH2891** | *rad55::ura4^+^, pfh1-R20, leu1-32, ade6-M210, ura4-D18, his3-D1,* Ch16-MGH, *h^+^* |
| **TH2906** | *rad55::arg3^+^*, *nbs1::ura4^+^, leu1-32, ade6-M210, ura4-D18, his3-D1,* arg3-D4, Ch16-MGH, *h+, pREP81X-HO* |
| **TH2953** | *rad55::ura4^+^, rqh1::rqh1(1-322D), leu1-32, ade6-M210, ura4-D18, his3-D1,* Ch16-MGH, *h^+^* |
| **TH2966** | *rad55::ura4^+^, rqh1::rqh1(1-322D), leu1-32, ade6-M210, ura4-D18, his3-D1,* Ch16-MGH, *h^+^*, *pREP81X-HO* |
| **TH2970** | *rad55::ura4^+^, pfh1-R20, leu1-32, ade6-M210, ura4-D18, his3-D1,* Ch16-MGH, *h^+^*, *pREP81X-HO* |
| **TH2999** | *rad51::ura4^+^*, *rad55::ura4^+^, rqh1::ura4^+^, leu1-32, ade6-M210, ura4-D18, his3-D1,* Ch16-MGH, *h^-^* |
| **TH3064** | *swi5::his3^+^*, *leu1-32, ade6-M210, ura4-D18, his3-D1,* Ch16-MGU, *h^+^* |
| **TH3034** | *rad51::ura4^+^*, *rad55::ura4^+^, rqh1::ura4^+^, leu1-32, ade6-M210, ura4-D18, his3-D1,* Ch16-MGH, *h^-^*, *pREP81X-HO* |
| **TH3044** | *swi5::his3^+^*, *rqh1::rqh1K547A, leu1-32, ade6-M210, ura4-D18, his3-D1* *arg3-D4,* Ch16-MGU, *h^-^* |
| **TH3064** | *swi5::his3^+^*, *leu1-32, ade6-M210, ura4-D18, his3-D1,* Ch16-MGU, *h^+^*, *pREP81X-HO* |
| **TH3079** | *swi5::his3^+^*, *rqh1::rqh1K547A, leu1-32, ade6-M210, ura4-D18, his3-D1* *arg3-D4,* Ch16-MGU, *h^-^*, *pREP81X-HO* |
| **TH3084** | *swi5::his3^+^*, *rad55::arg3^+^, leu1-32, ade6-M210, ura4-D18, his3-D1,* *arg3-D4* Ch16-MGU, *h^+^* |
| **TH3102** | *rad55::ura4^+^*, rqh*1::ura4^+^, leu1-32, ade6-M210, ura4-D18, his3-D1,* arg3-D4, Ch16-MGH, *h+, pIRT3* |
| **TH3103** | *swi5::his3^+^*, *rad55::arg3^+^, leu1-32, ade6-M210, ura4-D18, his3-D1,* *arg3-D4* Ch16-MGU, *h^+^*, *pREP81X-HO* |
| **TH3109** | *rad55::ura4^+^, rqh1::ura4^+^, leu1-32, ade6-M210, ura4-D18, his3-D1, arg3-D4* Ch16-MGH, *h^+^*, *pIRT3 -rad51* |
| **TH3110** | *rad55::ura4^+^, rqh1::ura4^+^, leu1-32, ade6-M210, ura4-D18, his3-D1, arg3-D4* Ch16-MGH, *h^+^*, *pREP81arg3-HO, pIRT3 -rad51* |
| **TH3120** | *rad55::ura4^+^, rqh1::ura4^+^, leu1-32, ade6-M210, ura4-D18, his3-D1, arg3-D4* Ch16-MGH, *h^+^*, *pREP81arg3-HO, pIRT3* |
| **TH3125** | *leu1-32, ade6-M210, ura4-D18, his3-D1,* arg3-D4, Ch16-MHH, *h+, pIRT3* |
| **TH3126** | *leu1-32, ade6-M210, ura4-D18, his3-D1,* arg3-D4, Ch16-MHH, *h+, pIRT3-Rad51* |
| **TH3175** | *swi5::his3^+^*, *rad55::arg3^+^, rqh1::rgh1K547A, leu1-32, ade6-M210, ura4-D18 his3-D1,* *arg3-D4,* Ch16-MGU, *h^+^* |
| **TH3185** | *swi5::his3^+^*, *rad55::arg3^+^, rqh1::rgh1K547A, leu1-32, ade6-M210, ura4-D18 his3-D1,* *arg3-D4,* Ch16-MGU, *h^+^*, *pREP81X-HO* |
| **TH3908** | *exo1::ura4^+^, rqh1::ura4^+^, ura4-D18, leu1-32, ade6-210, his3-D1,* Ch16-MGH |
| **TH3909** | *exo1::ura4^+^, rad55::ura4+, rqh1::ura4^+^, ura4-D18, leu1-32, ade6-210, his3-D1,* Ch16-MGH |
| **TH3945** | *exo1::ura4^+^*, *rqh1::ura4^+^, leu1-32, ade6-M210, ura4-D18, his3-D1,* Ch16-MGH, *h^-^*, *pREP81X-HO* |
| **TH3950** | *exo1::ura4^+^, rad55::ura4^+^, rqh1::ura4^+^, leu1-32, ade6-M210, ura4-D18 his3-D1,* Ch16-MGH, *h^+^*, *pREP81X-HO* |
| **TH8225** | *rqh1::ura4^+^, exo1::ura4^+^*, *ura4-D18, leu1-32, ade6-M210,* Ch16-MGTASTel |
| **TH8226** | *rqh1::ura4^+^, exo1::ura4^+^*, *ura4-D18, leu1-32, ade6-M210,* Ch16-MGTASTel, *pREP81X-HO* |
| **TH8413** | *ura4-D18, leu1-32, ade6-M210,* Ch16-MG(TASTel)Ch, (Ch16 *ade6-M216 rad21::MATa kanMX6* TASTel Ch16) *h^-^* |
| **TH8597** | *ura4-D18, leu1-32, ade6-M210,* Ch16-MG(TASTel)Ch, *h^-^*, *pREP81X-HO* |
| **TH8598** | *rqh1::ura4^+^, exo1::ura4^+^*, *ura4-D18, leu1-32, ade6-M210,* Ch16-MG(TASTel)Ch, *pREP81X-HO* |
| **TH8708** | *rqh1::ura4^+^, rad55::ura4^+^*, *ura4-D18, leu1-32, ade6-M210,* Ch16-MG(TASTel)Ch, *pREP81X-HO* |
| **TH8919** | *pfh1-R20, leu1-32, ade6-M210, ura4-D18, his3-D1,* Ch16-MGH, *h+, pREP81X-HO* |
| **TH9207** | *rad55::arg^+^, leu1-32, ade6-M210, ura4-D18, his3-D1, arg3-D4* Ch16-MGH, *h^+^*, *pIRT3* |
| **TH9210** | *rad55::arg^+^, leu1-32, ade6-M210, ura4-D18, his3-D1, arg3-D4* Ch16-MGH, *h^+^*, *pIRT3 -rad51* |
| **TH9213** | *rqh1::ura4^+^, leu1-32, ade6-M210, ura4-D18, his3-D1,* Ch16-MGH, *h^+^*, *pIRT3* |
| **TH9216** | *rqh1::ura4^+^, leu1-32, ade6-M210, ura4-D18, his3-D1,* Ch16-MGH, *h^+^*, *pIRT3-rad51* |

**Figure S1**

**Spot dilution assays for viability after DSB induction**


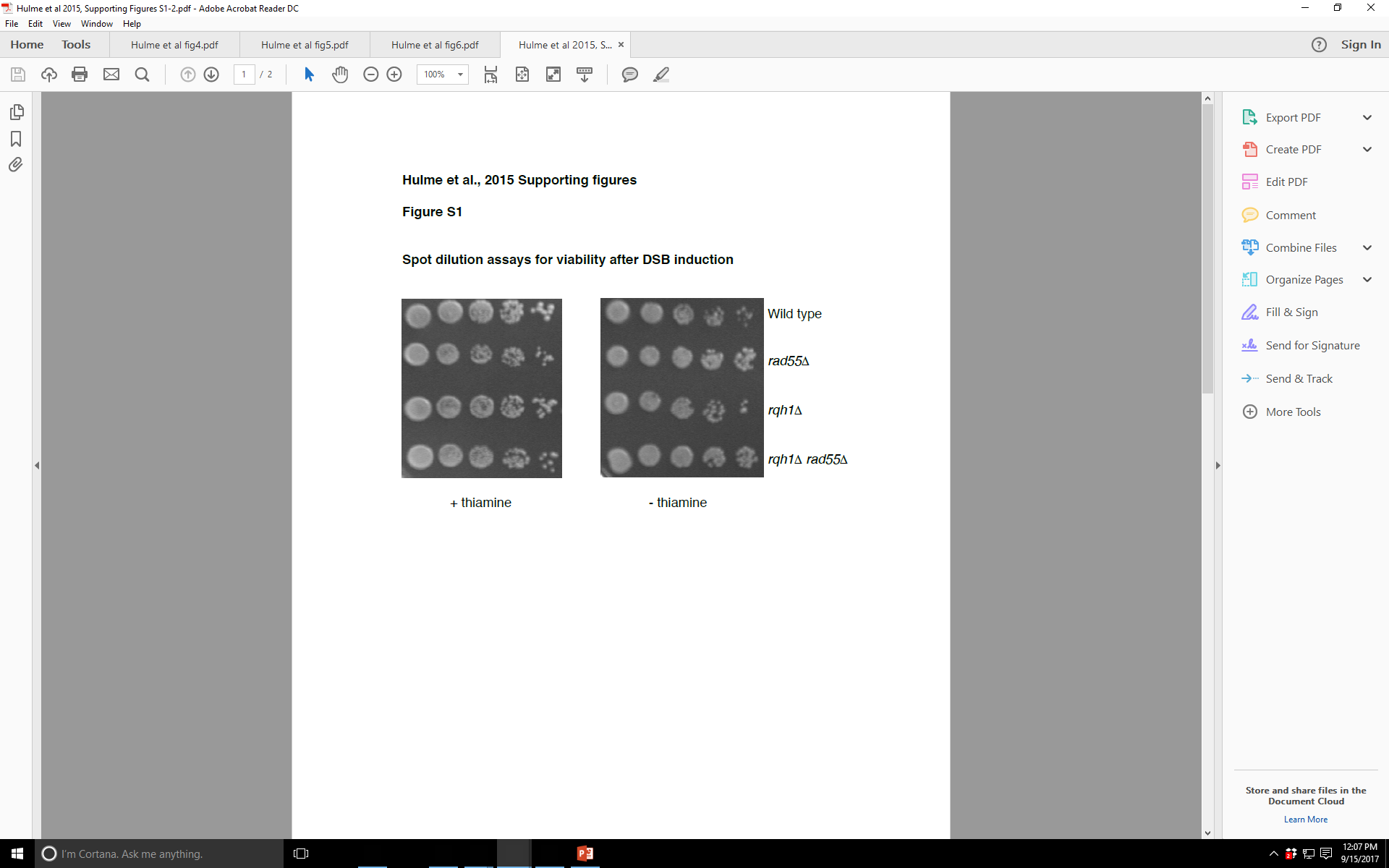


**Figure S2**

**Radiation sensitivity of *rad55Δrqh1Δ* in the context of *rad51Δ***

***
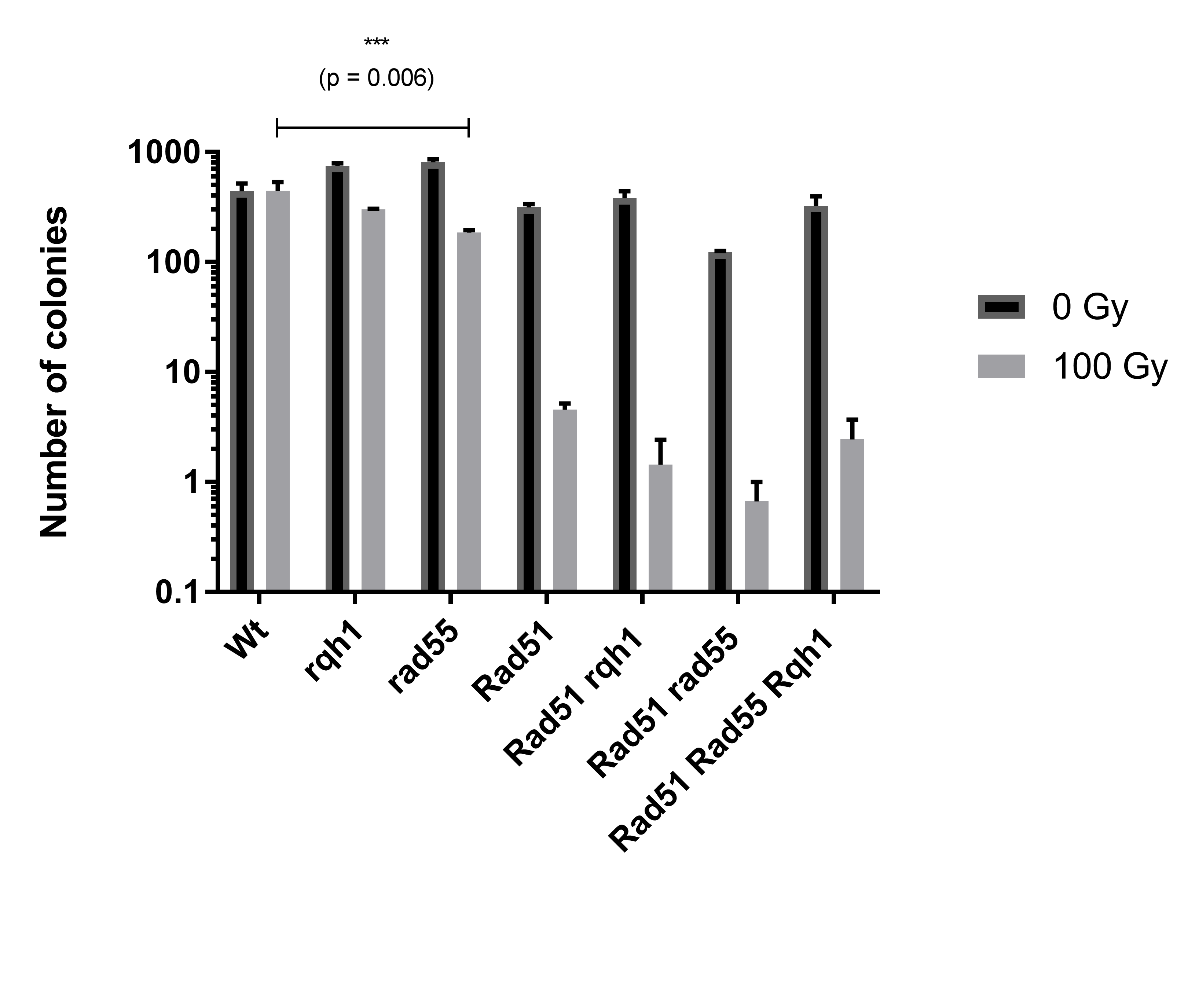
***

**Figure S3**

**Radiation sensitivity of *rad55Δrqh1Δ* in the context of Rad51^+^ over-expression**

***
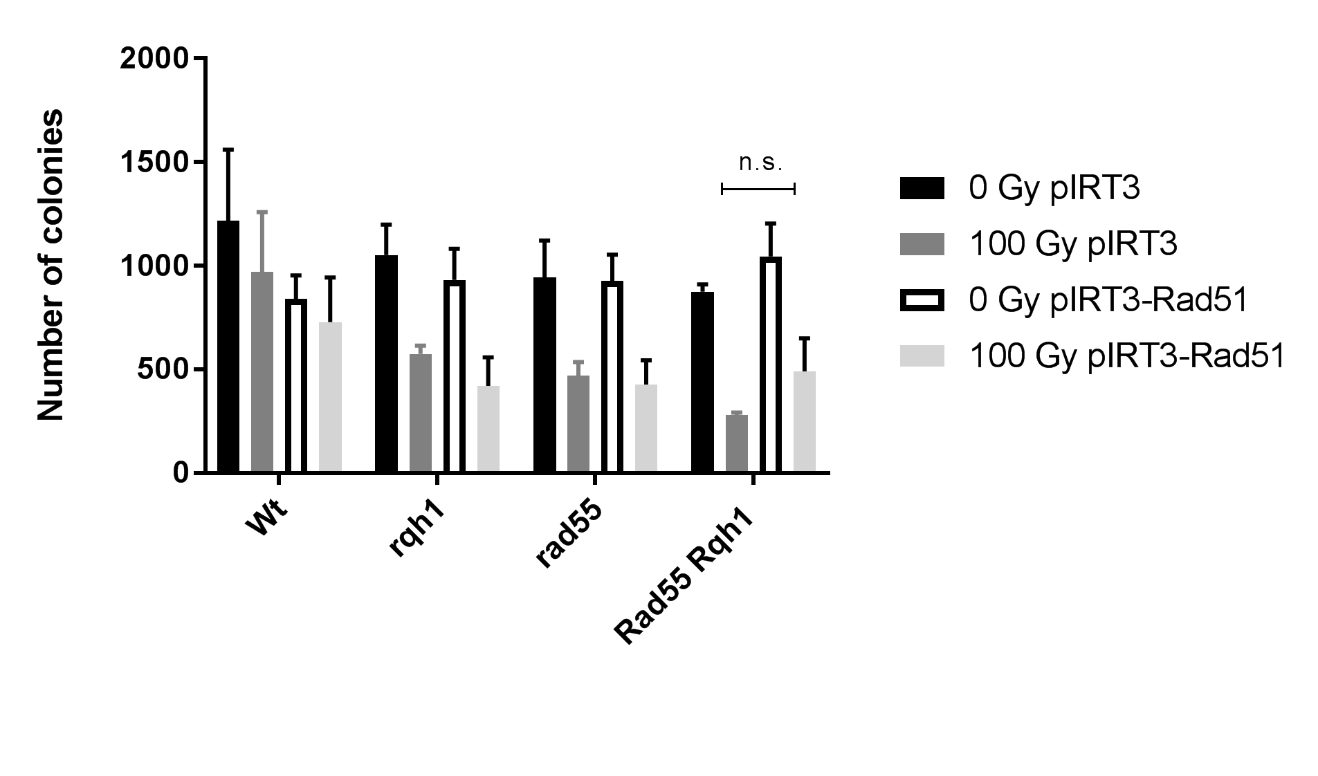
***
